# Taurine attenuates polystyrene microplastic–associated oxidative stress, inflammation, and apoptotic changes in the spleen of mice

**DOI:** 10.3389/fphar.2026.1820042

**Published:** 2026-06-25

**Authors:** Lujin A. Essa, Rawan Altalhi, Nouf M. Alshehri, Amany Abdel-Rahman Mohamed, Manal E. Alosaimi, Ahmed E. Noreldin, Tarek Khamis, Eman K. Rashwan, Yasmina M. Abd-Elhakim

**Affiliations:** 1 Department of Biological Sciences, College of Science, University of Jeddah, Jeddah, Saudi Arabia; 2 Department of Forensic Medicine and Toxicology, Faculty of Veterinary Medicine, Zagazig University, Zagazig, Egypt; 3 Department of Basic Sciences, College of Medicine, Princess Nourah bint Abdulrahman University, Riyadh, Saudi Arabia; 4 Histology and Cytology Department, Faculty of Veterinary Medicine, Damanhour University, Damanhour, Egypt; 5 Pharmacology Department, Faculty of Veterinary Medicine, Zagazig University, Zagazig, Egypt; 6 Laboratory of Biotechnology, Faculty of Veterinary Medicine, Zagazig University, Zagazig, Egypt; 7 Department of Physiology, College of Medicine, Al-Azhar University, Assiut, Egypt; 8 Department of Physiology, College of Medicine, Jouf University, Sakaka, Saudi Arabia

**Keywords:** caspase-3, COX-2, immunotoxicity, inflammation, microplastics, NF-κB, oxidative stress, taurine

## Abstract

**Introduction:**

Microplastic (MP) contamination is an emerging environmental threat with potential adverse effects on oxidative stress, immune function, and inflammatory homeostasis. This study investigated the oxidative stress, pro-inflammatory, and immunotoxic effects of polystyrene microplastics (PMPs) and evaluated the protective role of the nutraceutical taurine (TN) in male Swiss mice following 60 days of oral exposure.

**Methods:**

Mice were assigned to four groups: control, PMPs (10 mg/kg b.wt.), TN-treated (200 mg/kg b.wt.), and PMPs + TN. Hematological parameters and serum immune markers, including immunoglobulins G and M (IgG and IgM), complement component 3 (C3), and nitric oxide (NO), were assessed. Splenic tissue was analyzed for oxidative stress markers, including malondialdehyde (MDA), catalase (CAT), and superoxide dismutase (SOD); pro-inflammatory mediators, including tumor necrosis factor-alpha (TNF-α), interleukin-1 beta (IL-1β), and nuclear factor kappa B (NF-κB); apoptotic markers, including caspase-3 and B-cell lymphoma 2 (BCL-2); and immune-regulatory genes, including cluster of differentiation 4 and 8 (CD4 and CD8). Histopathology (hematoxylin and eosin (H&E) and periodic acid–Schiff (PAS) staining) and immunohistochemistry for cyclooxygenase-2 (COX-2) and caspase-3 were performed to evaluate structural alterations and inflammatory/apoptotic signaling in the spleen.

**Results:**

PMPs exposure induced significant hematological disturbances, systemic inflammation, immune alterations, and elevated NO levels. In splenic tissue, PMPs caused oxidative stress and inflammation, evidenced by increased MDA, and TNF-α levels and reduced CAT and SOD activities. Histological and immunohistochemical analyses revealed structural splenic damage with enhanced Caspase-3 and COX-2 expression, indicating elevated apoptosis and inflammatory signaling. Gene expression analysis revealed upregulation of IL-1β, TNF-α, NF-κB, and Caspase-3, and CD8, wheras BCL-2 and CD4 were significantly downregulated. Taurine supplementation effectively mitigated PMPs-induced effects by restoring hematological, alleviating immune alterations, enhancing antioxidant defenses, reducing inflammatory and apoptotic markers, and improving gene expression profiles.

**Conclusion:**

These findings demonstrated that TN exerts protective effects against PMPs-induced oxidative stress, inflammation, apoptosis, and immune-related alterations in splenic tissue. TN partially attenuated PMPs-induced immunotoxic and histopathological changes in this experimental mouse model, although further mechanistic, dose-response, and functional immune studies are required to confirm its potential therapeutic or nutraceutical applications against PMPs -induced toxicity.

## 1 Introduction

Polystyrene microplastics (PMPs) originate from multiple sources, comprising the degradation of larger plastic materials, industrial activities, personal care products, and synthetic textiles (Akanyange et al., 2022; Kochanek et al., 2025). The fragmentation of larger plastic debris and the extensive use of polystyrene in consumer products substantially contribute to their environmental abundance (Kong et al., 2025). As a result, PMPs are now ubiquitous in the environment and have been detected in air, water, soil, and food sources (Men et al., 2025; Salih et al., 2026), with global distribution ranging from remote regions such as the Arctic to densely populated urban areas (An et al., 2020). Human exposure to PMPs occurs mainly through the consumption of contaminated drinking water and food, inhalation of airborne particles, particularly in urban environments, and, to a lesser extent, dermal contact with contaminated water or consumer products (Yadav et al., 2022; Krishnan, 2023). Growing evidence has linked PMPs exposure to various adverse health outcomes, including cardiovascular disease (Du et al., 2025), respiratory disorders (Balkrishna et al., 2025; Du et al., 2026), neurobehavioral disturbances (Wang et al., 2024), gut microbiota dysbiosis (Le et al., 2026), disruption of hepatic lipid metabolism (Tian et al., 2025), and certain types of cancer (Lee et al., 2025; Li et al., 2025). Among the affected biological systems, the immune system has emerged as a critical target of PMPs’ toxicity.

The immune system may distinguish PMPs as foreign substances, triggering immune responses that can lead to chronic inflammation and immune alterations (Yang et al., 2022). Experimental studies have shown that PMPs can induce immunosuppression by reducing spleen weight and decreasing cluster of differentiation 8 (CD8) T-cell populations in the spleen of mice (Wang J. et al., 2022). In addition, mouse neutrophils have been shown to bind and engulf polystyrene PMPs, resulting in a proinflammatory state and apoptotic cell death mediated through toll-like receptor pathways (Park et al., 2024), suggesting that PMPs may mimic bacterial recognition and exacerbate inflammatory responses. PMPs have also been reported to reduce the viability of immune cells such as macrophages and leukocytes and impair key immune functions, including phagocytosis and cytokine production (Koner et al., 2023; Shitikova et al., 2024; Koner et al., 2025). Furthermore, exposure to PMPs induces oxidative stress and inflammatory responses in immune cells, leading to cellular damage and dysfunction (Zhang Q. et al., 2024). Microplastics may upregulate proinflammatory cytokines such as interleukin-1α in serum, reduce regulatory T (Treg) and IL-17–producing T helper cells among splenic cluster of differentiation 4 (CD4) T cells, and evoke intestinal inflammation via activation of toll-like receptor pathways (Li et al., 2020). Chronic exposure to PMPs has therefore been associated with immune perturbations, altered cytokine expression, and increased susceptibility to infections (Maione et al., 2024; Shang et al., 2024).

In addition to immune toxicity, PMPs exposure has been linked to hematological disturbances. Exposure of mice to high concentrations of PMPs (6, 60, and 600 μg/day) for 15 days resulted in abnormal erythrocyte morphology, contributing to anemic patterns (Abdel-Zaher et al., 2023). Several studies have reported reductions in red blood cell (RBCs) count and hemoglobin (Hb) levels following PMPs exposure, indicating anemia in rats administered varying concentrations of PMPs solutions (Abdel-Zaher et al., 2023; Razzaq et al., 2024; Yahaya et al., 2024). Additionally, a reduction in platelet count accompanied by increases in mean corpuscular hemoglobin (MCH) and mean corpuscular volume (MCV) was observed in mice treated with polyethylene microfibers at doses of 100–800 μg/day for 35 days (Razzaq et al., 2024). A decrease in white blood cells (WBCs) count, indicative of immunosuppressive effects, has also been documented, particularly in mice exposed to PMPs (Abdel-Zaher et al., 2023). Morphological alterations in immune-related organs have also been reported, including inflammatory infiltration and changes in goblet cell volume fraction in the colon mucosa of mice administered 5-μm PMPs for 4 weeks at doses ranging from 0.023 to 2.3 mg/kg/day (Zolotova et al., 2023). Similarly, exposure to 5-μm PMPs at doses of 0.1 and 0.5 mg resulted in reduced WBCs counts and altered immune-related gene expression in bone marrow cells (Sun et al., 2021).

In light of these findings, increasing attention has been directed toward potential protective agents capable of mitigating PMPs-induced immunotoxicity. Taurine (TN) has gained increasing research attention because of its diverse roles in numerous physiological functions and its promising therapeutic and health-promoting properties (Zhang C. et al., 2024). Taurine is a sulfur-containing amino acid that plays a key role in immune modulation through multiple mechanisms (Sartori et al., 2018; Fang et al., 2019). It has been shown to influence the expression of immune-related genes in macrophages by significantly increasing the mRNA levels of chemokine receptors such as CXCR2 and CXCR4, which are essential for immune cell function (Satsu et al., 2022). Taurine also enhances B-cell receptor–mediated signaling, resulting in increased B-cell activation and immunoglobulin G (IgG) production, thereby supporting adaptive immunity (Song et al., 2023). Moreover, TN modulates the activity of regulatory T cells, which are essential for sustaining immune tolerance and inhibiting autoimmune responses. For example, TN pretreatment in a rat model of mastitis increased the proportion of Tregs, leading to reduced inflammation and tissue damage (Miao et al., 2012). Taurine further exerts anti-inflammatory effects by downregulating the production of proinflammatory mediators, including nitric oxide (NO), superoxide anion, tumor necrosis factor-α (TNF-α), prostaglandins, and interleukins in inflammatory cells (Kim and Cha, 2014). Taurine chloramine (TauCl), a derivative formed during inflammatory responses, inhibits the activation of nuclear factor kappa-light-chain-enhancer of activated B cells (NF-κB), a central regulator of inflammation, thus decreasing proinflammatory cytokine production (Schuller-Levis and Park, 2004; Kim and Cha, 2014). TauCl also contributes to inflammation resolution by enhancing antioxidant protein expression and reducing oxidative stress (Kim and Cha, 2014). Therefore, the present study aimed to investigate whether TN could mitigate the immunotoxic effects induced by PMPs exposure in mice, which are hypothesized to occur through immune modulation, oxidative stress, and apoptotic pathways. To test this, mice exposed to PMPs and/or TN were subjected to comprehensive biochemical, histopathological, immunohistochemical, and molecular analyses.

## 2 Materials and methods

### 2.1 Chemicals, PMPs preparation, and experimental animals

Taurine (≥98% purity) was procured from Alfa Chemistry (Holbrook, NY, USA) with Catalog No. ACM107357-5. Polystyrene microplastics were synthesized using a suspension polymerization method, as previously described in our recent study Eskandrani et al. (2025). The chemical composition and functional groups were confirmed by attenuated total reflectance Fourier-transform infrared spectroscopy (ATR-FTIR), which showed characteristic aromatic and aliphatic C–H stretching vibrations and C=C aromatic ring peaks, indicating complete polymerization. Particle morphology and size were examined using scanning electron microscopy (SEM), which revealed predominantly spherical particles with smooth surfaces and a narrow size distribution. Hydrodynamic diameter measured by dynamic light scattering (DLS) confirmed an average particle size of approximately 1.8 µm and a low polydispersity index (PDI = 0.133), indicating uniformity. To ensure a uniform suspension and minimize aggregation prior to oral administration, the PMPs stock solution was freshly dispersed in distilled water and gently stirred on a magnetic stirrer immediately before dosing. Eighty male Swiss albino mice (6 weeks of age; 20–25 g) were obtained from the Laboratory Animal Housing Unit, Faculty of Veterinary Medicine, Zagazig University, Egypt. Animals were maintained under controlled environmental conditions (12 h light/dark cycle, adequate ventilation, and standard temperature and humidity) with unrestricted access to commercial rodent chow and water. A 14-day acclimatization period was allowed before initiation of the experimental procedures to ensure physiological stabilization.

### 2.2 Ethical compliance and study design

All experimental procedures were approved by the Institutional Animal Care and Use Committee (IACUC), Faculty of Veterinary Medicine, Zagazig University (Approval No. ZU-IACUC/2/F/33/2025). The study was performed in accordance with the United Kingdom. Animals (Scientific Procedures) Act, the European Union Directive 2010/63/EU, and the Guide for the Care and Use of Laboratory Animals. Reporting standards complied with the ARRIVE guidelines (Du Sert et al., 2020). Animals were randomly distributed into four equal groups (n = 20 per group): Control group: received 0.5 mL of distilled water orally. TN-treated group: administered TN at 200 mg/kg b. wt (Niu et al., 2018; Song et al., 2024). PMPs-exposed group: received 10 mg/kg b.wt PMPs suspended in distilled water. Combined PMPs + TN treatment group: co-treated with PMPs and TN at the above-mentioned doses. All treatments were delivered orally once daily for 60 consecutive days. Body mass was measured weekly to adjust administered doses. Throughout the experiment, animals were observed for clinical abnormalities, including behavioral changes, respiratory disturbances, mucosal discoloration, signs of illness, or mortality.

### 2.3 PMPs and TN dose selection

The dose of PMPs administered in mice (10 mg/kg b.wt.) was selected to simulate oral exposure, which represents the primary route of human PMPs intake through contaminated food and drinking water (Pironti et al., 2021; Adikari et al., 2023; Zhu et al., 2024). Previous studies have reported that humans are continuously exposed to measurable quantities of PMPs through dietary and environmental sources in the range of 0.22–0.66 mg/kg b.wt (Cox et al., 2019; Fadare et al., 2020; Deng et al., 2022). To provide translational context, body surface area (BSA)-based dose conversion was considered according to the method described by Nair and Jacob (2016). Using this conventional scaling approach, the administered mouse dose approximately corresponds to 0.8 mg/kg b.wt in humans. Although BSA-based interspecies scaling is commonly used for conventional pharmacological agents, its direct application to particulate materials such as PMPs should be interpreted cautiously because PMPs may differ substantially from soluble compounds in gastrointestinal absorption, biodistribution, tissue accumulation, and clearance characteristics (Hirt and Body-Malapel, 2020; Wu et al., 2022). Nevertheless, the selected dose falls within the range of experimentally utilized exposure levels reported in previous mice studies investigating the toxicological effects of PMPs. Several experimental studies have employed oral PMPs doses of approximately 10 mg/kg b.wt to evaluate oxidative stress, apoptotic reactions, inflammatory responses, hepatotoxicity, and reproductive toxicity in mice under sub-chronic exposure conditions (Lu et al., 2023; Jiang et al., 2025). Therefore, the dose of 10 mg/kg b.wt was considered appropriate for investigating the oral toxicological and immunomodulatory effects of PMPs under controlled experimental conditions.

The dose of TN used in this study (200 mg/kg b.wt) was selected based on previous murine studies demonstrating its antioxidant, anti-inflammatory, and cytoprotective effects across a range of experimental conditions. Iliya (2015) evaluated TN at doses of 100, 200, and 400 mg/kg b.wt in restraint stress models, whereas Jang et al. (2017) used 250 mg/kg b.wt/day in an Alzheimer’s disease model. Likewise, studies examining dietary challenges and heat-stress conditions demonstrated that TN doses between 100 and 200 mg/kg b.wt exerted substantial antioxidant and tissue-protective effects while remaining well tolerated and free of major adverse effects (Wu et al., 2013; Yu et al., 2016; Doss et al., 2022; Ren et al., 2026). Based on these findings, 200 mg/kg b.wt was selected as an effective and safe intermediate dose within the reported therapeutic range to evaluate its potential protective effects against PMPs-induced splenic injury.

### 2.4 Sample collection and tissue preparation

At study termination (day 60), a randomly selected subset of six mice per group (n = 6) was anesthetized with isoflurane and humanely euthanized. To ensure randomization and minimize selection bias, all animals within each experimental group were assigned numerical identification codes, and mice were selected using a computer-generated randomization sequence (Microsoft Excel RAND function) by an investigator not involved in downstream analyses, in accordance with ARRIVE guidelines recommendations (Du Sert et al., 2020). In addition, all animals were age-matched, housed under identical environmental conditions, and subjected to the same handling and experimental procedures throughout the study period to minimize potential confounding variables. The same randomly selected subset of animals was consistently used across all hematological, biochemical, molecular, histopathological, and immunohistochemical assessments. Blood was collected via the retro-orbital plexus. Samples designated for hematological evaluation were transferred into EDTA-containing tubes, whereas samples for serum separation were allowed to clot in plain tubes. Serum was obtained by centrifugation (3,000 rpm, 10 min) and stored at −20 °C until biochemical assessment. Spleens were excised immediately, rinsed in ice-cold saline, and subdivided into portions allocated for: Biochemical assays: homogenized at 4 °C (15 min, 664 × g), Histological and immunohistochemical studies: fixed in 10% neutral-buffered formalin, Molecular investigations: preserved at −80 °C for RNA extraction and gene expression analysis.

### 2.5 Hematological evaluation

Complete blood counts, including RBCs count, Hb concentration, hematocrit (HCT), MCV, MCH, mean corpuscular hemoglobin concentration (MCHC), total WBCs count, and leukocyte differential, were measured using an automated hematology analyzer (HemaScreen 18, Hospitex Diagnostics, Italy) in line with Feldman et al. (2000) protocol. Differential counts were verified through microscopic examination of stained blood smears following standard hematological procedures (Dacie JV, 1991).

### 2.6 Determination of serum immune markers

Serum levels of immunoglobulin M (IgM), IgG, and complement component 3 (C3) were quantified using commercially available ELISA kits (MyBioSource, San Diego, CA, USA) according to the manufacturer’s instructions. Samples were diluted where appropriate to fit within the specified detection ranges. The ELISA kits employed in this study and their analytical specifications were as follows: the IgG kit (Cat. No. MBS702698) exhibited a measurable range of 125–8,000 ng/mL with an assay sensitivity below 29 ng/mL; the IgM kit (Cat. No. MBS703424) demonstrated a quantification interval of 62.5–4,000 ng/mL and a sensitivity threshold of less than 62.5 ng/mL; and the complement C3 kit (Cat. No. MBS2513804) covered a detection span of 3.125–200 ng/mL with a minimum detectable concentration of 1.88 ng/mL.

### 2.7 Evaluation of oxidative stress and inflammatory markers

Spleen tissue homogenates were prepared and subjected to biochemical analysis to determine oxidative stress status. Lipid peroxidation was estimated by measuring malondialdehyde (MDA) levels, while antioxidant defense parameters, including superoxide dismutase (SOD) activity and catalase (CAT) activity, were assessed using commercially available assay kits supplied by Biodiagnostic Co. (Egypt) (Cat. Nos. MD 25 29, SD 25 21, and CA 25 17, respectively). All assays were performed in accordance with the manufacturer’s standardized instructions. In addition, splenic concentrations of TNF-α and NO were determined using a quantitative ELISA kit (MyBioSource, San Diego, CA, USA; Cat. No. MBS825075 and MBS2540417, respectively), which provides a detection range of 15.6–1000 pg/mL and 0.97–700 μmol/L and a sensitivity below 8 pg/mL and 0.97 μmol/L, respectively. Analytical procedures were strictly conducted following the guidelines provided by the respective manufacturers.

### 2.8 Histopathological and histochemical examination

Formalin-fixed splenic tissues were processed routinely, embedded in paraffin, sectioned at 4 μm, and stained with hematoxylin and eosin (H&E) along with the procedure defined by Bancroft and Layton (2013). Histological alterations were assessed semi-quantitatively using a standardized scoring system of Gibson-Corley et al. (2013). Lesions were graded on a scale from 0 (normal) to 4 (76%–100% tissue involvement).

Periodic acid–Schiff (PAS) staining was performed to evaluate PAS-reactive carbohydrate-rich components, including glycogen, glycoproteins, and other polysaccharide-containing structures within the splenic tissue (Mahmoudi Aliabadi et al., 2024). Quantitative image analysis was conducted using Fiji ImageJ software (National Institutes of Health, Bethesda, MD, USA). The images were subjected to color deconvolution to quantify PAS-positive areas (magenta), with consistent threshold parameters applied across all samples (Vis et al., 2000; Schindelin et al., 2012). All quantitative analyses were performed on a randomly selected subset of six animals per group (n = 6). For each animal, ten non-overlapping microscopic fields per region were examined at ×40 magnification, and these fields were treated as technical replicates and averaged to yield a single value per animal.

### 2.9 Immunohistochemical examination

Immunohistochemical detection was carried out to evaluate the expression of cleaved caspase-3 and cyclooxygenase-2 (COX-2) in splenic tissue sections. A polyclonal rabbit anti-cleaved caspase-3 antibody (1:100 dilution; BioCare Medical, Cat. No. CP229C, Concord, CA, USA) and a monoclonal rabbit anti-COX-2 antibody (1:100 dilution; Thermo Fisher Scientific, Cat. No. RM-9121-S0, Fremont, CA, USA) were employed as primary antibodies. Heat-induced antigen retrieval was performed solely for COX-2 staining using 10 mM citrate buffer (pH 6.0) at 105 °C for 20 min. The staining procedure was implemented in accordance with the protocol described by Saafan et al. (2023). Stained sections were examined microscopically, and representative photomicrographs were obtained using a Leica EC3 digital imaging system (Leica Microsystems, Germany).

Quantitative evaluation of immunoreactivity was conducted using Fiji ImageJ software (National Institutes of Health, Bethesda, MD, USA) following validated analytical workflows (Schindelin et al., 2012; Buckels et al., 2022). For each biomarker, ten randomly selected, non-overlapping microscopic fields per animal were analyzed and averaged to generate a single value, with six animals included in each group. Color deconvolution was applied to selectively extract the brown chromogenic signal corresponding to positive staining. Consistent threshold parameters were maintained across all images to determine the percentage area of immunopositive staining, as previously outlined in established image analysis methodologies (Vis et al., 2000).

### 2.10 Quantitative real-time PCR (qRT-PCR)

Gene expression profiling was performed to assess immune, inflammatory, and apoptotic markers, comprising CD4, CD8, interleukin-1 beta (IL-1β), TNF-α, NF-κB-p65, caspase-3 (Casp-3), and B-cell lymphoma-2 (BCL-2). Total RNA was isolated from splenic tissue by TRIzol™ reagent (Invitrogen, Thermo Fisher Scientific, USA), treated with DNase I, and purified using RNeasy spin columns (QIAGEN). RNA concentration and purity were evaluated spectrophotometrically (NanoDrop™ One, Thermo Fisher Scientific). Complementary DNA (cDNA) synthesis was followed by amplification using SYBR® Green chemistry on a Rotor-Gene Q platform (Qiagen, Germany). Primer specificity was confirmed through melt-curve analysis and agarose gel electrophoresis. Amplification efficiency (95%–104%) was confirmed using standard curves derived from serial dilutions. No-template controls were included in each run. The nucleotide sequences of all primers are provided in Table 1. Relative transcript levels were normalized to glyceraldehyde-3-phosphate dehydrogenase (GAPDH), identified as the most stable housekeeping gene using the geNorm algorithm (Vandesompele et al., 2002). Quantitative PCR reactions were performed by a QuantStudio™ 6 Flex Real-Time PCR System (Applied Biosystems, USA). Fold changes in gene expression were calculated according to the 2^^−ΔΔCt^ method with efficiency correction (Schmittgen and Livak, 2008).

**TABLE 1:** Oligonucleotide primer sequences and real-time PCR conditions.

Gene	Primers sequence		Size	Accession no.
CD4	F	CAC​ACA​CCT​GTG​CAA​GAA​GC	169	NM_013488.3
	R	GCG​TCT​TCC​CTT​GAG​TGA​CA		
CD8	F	GGA​TTG​GAC​TTC​GCC​TGT​GA	129	NM_001081110.2
	R	GGG​ACA​TTT​GCA​AAC​ACG​CT		
IL-1β	F	GCC​ACC​TTT​TGA​CAG​TGA​TGA​G	163	NM_008361.4
	R	TGA​TAC​TGC​CTG​CCT​GAA​GC		
TNF-α	F	ACT​GAA​CTT​CGG​GGT​GAT​CG	99	NM_001278601.1
	R	TGG​TTT​GTG​AGT​GTG​AGG​GT		
NF-KB-p65	F	AGA​TGT​TCA​CGG​TGT​GAC​CC	181	NM_001402548.1
	R	ACA​GGC​CTT​AGG​GTA​GAG​GG		
Casp-3	F	GGG​GAG​CTT​GGA​ACG​CTA​AG	116	NM_009810.3
	R	GAG​TCC​ACT​GAC​TTG​CTC​CC		
BCL-2	F	GAA​CTG​GGG​GAG​GAT​TGT​GG	194	NM_009741.5
	R	GCA​TGC​TGG​GGC​CAT​ATA​GT		
Gapdh	F	TTCTCCTGCAGCCTCGT	178	NM_001411843.1
	R	ACG​GCC​AAA​TCT​TGA​GGT​CT		

CD4: Cluster of differentiation 4; CD8: Cluster of differentiation 8; IL-1β: Interleukin-1, beta; TNF-α: tumor necrosis factor alpha; NF-κB-p65: Nuclear factor kappa-light-chain-enhancer of activated B cells p65 subunit; Casp-3: Caspase-3; BCL-2: B-cell lymphoma-2; GAPDH: Glyceraldehyde-3-phosphate dehydrogenase.

### 2.11 Statistical analysis

Data distribution and variance homogeneity were evaluated using Kolmogorov–Smirnov and Levene’s tests, respectively. Parametric data were analyzed by one-way ANOVA followed by Tukey’s multiple comparison test by IBM SPSS Statistics (version 21, IBM Corp., Armonk, NY, USA). Non-parametric histopathological scores were assessed with the Kruskal–Wallis test followed by Dunn’s *post hoc* test. Each analysis was performed using six independent biological replicates per experimental group, whereas technical replicates were averaged and not considered independent observations for statistical purposes. Effect sizes were determined for all primary comparisons, and 95% confidence intervals were provided where appropriate. In addition, *post hoc* power analysis using one-way ANOVA (α = 0.05), based on the observed effect sizes, demonstrated a statistical power of ≥0.95 for all major endpoints, indicating that the selected sample size was adequate to detect biologically meaningful differences. Results are expressed as mean ± standard error (SE), with statistical significance set at *P* < 0.05. Graphical representations were generated by GraphPad Prism (version 8, GraphPad Software Inc., San Diego, CA, USA).

Principal correlation analysis was performed using all biological replicates obtained from the same randomly selected animals included in the experimental measurements (n = 6 animals per group; total n = 24). Each animal was treated as a single independent biological observation, and no technical replicates were considered independent data points. All variables included in the multivariate analyses followed a normal distribution; therefore, no data transformation was applied. Correlation analyses were conducted using matched measurements from the same individual animals across all experimental groups to evaluate associations among hematological indices, serum immune parameters, oxidative stress markers, antioxidant enzymes, inflammatory cytokines, apoptotic markers, immune-regulatory genes, histopathological PAS-positive area percentages, and immunohistochemical markers. The analysis incorporated blood indices (Hb content, RBCs, platelets, WBCs, lymphocyte, and neutrophil counts), serum immune parameters (IgG, IgM, complement C3, and NO), splenic oxidative stress markers (MDA, CAT, and SOD), pro-inflammatory cytokine expression (TNF-α, IL-1β, and NF-κB), apoptotic and immune-regulatory gene expression (Caspase-3, BCL-2, CD4, and CD8), and immunohistochemical markers (Caspase-3 and COX-2). Correlation coefficients were visualized using a Pearson correlation heatmap following the method described by Granato et al. (2018) by treating each animal as a single independent biological replicate.

## 3 Results

### 3.1 Effects of PMPs and/or TN on hematological indices

As presented in Table 2, oral exposure of adult male Swiss mice to PMPs for 60 days induced marked alterations in erythrogram and leukogram parameters compared with the control group. PMPs exposure significantly (*P* < 0.001) increased RBCs count, Hb, and HCT by 82.3%, 90.1%, and 69.0%, respectively, relative to controls. MCHC were also significantly (*P* < 0.001) elevated by 11.9%. MCH and MCV were not significantly affected. Platelet count exhibited a significant (*P* < 0.001) thrombocytosis, rising by 344.7% compared with control values. Concomitantly, PMPs exposure significantly (*P* < 0.001) suppressed WBCs by 47.9%, accompanied by a marked neutrophilia (+157.2%) and basophilia (+65.2%), along with significant reductions in lymphocytes (−24.4%) and monocytes (−46.7%). Eosinophils and band neutrophils were not significantly affected among groups.

**TABLE 2:** Effect of taurine (TN) oral dosing on hematological indices of adult male Swiss mice exposed to polystyrene microplastics (PMPs) for 60 days.

Estimated parameters	C	TN	PMPs	PMPs + TN
Erythrogram				
RBCs (10^6^/mm^3^)	2.94 ± 0.02	2.78 ± 0.06	5.36*** ± 0.21	3.14 ± 0.11
Hb (g/dL)	7.97 ± 0.16	7.61 ± 0.05	15.15*** ± 0.22	9.18***^###^ ±0.11
HCT (%)	22.60 ± 0.96	21.86 ± 0.63	38.20*** ± 0.25	25.26^###^ ±0.70
MCV(fl)	76.74 ± 2.90	79.03 ± 3.39	71.70 ± 2.62	81.11 ± 4.62
MCH (%)	27.06 ± 0.40	27.46 ± 0.66	28.40 ± 0.84	29.35 ± 0.85
MCHC (%)	35.44 ± 1.02	34.92 ± 0.96	39.67* ± 0.35	36.50 ± 1.14
Platelets	181.81 ± 5.22	175.78 ± 4.27	808.47*** ± 26.76	355.04***^###^ ± 19.41
Leukogram				
WBCs (10^3^/mm^3^)	14.74 ± 0.38	16.31 ± 0.57	7.68*** ± 0.29	12.20**^###^ ± 0.48
Segmented neutrophils (%)	13.80 ± 1.00	13.17 ± 0.70	35.50*** ± 0.59	21.26***^###^ ± 1.05
Lymphocytes (%)	79.75 ± 1.30	80.88 ± 0.73	60.28*** ± 0.58	73.29***^###^ ±0.96
Eosinophils (%)	0.27 ± 0.01	0.25 ± 0.01	0.25 ± 0.01	0.23 ± 0.02
Stab (band) neutrophils (%)	0.81 ± 0.00	0.80 ± 0.02	0.81 ± 0.01	0.78 ± 0.02
Monocytes (%)	5.18 ± 0.02	5.34 ± 0.27	2.76 *** ± 0.03	4.17 ***^###^ ± 0.08
Basophils (%)	0.23 ± 0.01	0.20 ± 0.01	0.38*** ± 0.01	0.29**^##^± 0.01

RBCs: red blood cells; Hb: hemoglobin; HCT: hematocrit; MCV: mean corpuscular volume; MCH: mean corpuscular hemoglobin; MCHC: mean corpuscular hemoglobin concentration; WBC: white blood cells. Data are presented as mean ± SE (n = 6 per group). Statistical significance is indicated as: *** (*P* < 0.001), ** (*P* < 0.01), and *(*P* < 0.05) vs. control and ### (*P* < 0.001) and ## (*P* < 0.01) vs. PMPs group. One-way ANOVA with Tukey’s *post hoc* test was used for comparisons.

Relative to the PMPs group, co-administration of TN with PMPs significantly (*P* < 0.001) reduced RBCs, Hb, and HCT by 41.4%, 39.4%, and 33.9%, respectively, restoring these parameters toward control levels. Platelet count was significantly (*P* < 0.001) decreased (−56.1%) compared with PMPs-exposed mice. In the leukogram, TN co-treatment significantly (*P* < 0.001) increased total WBCs by 58.9% compared with the PMPs group. This effect was associated with a reduction in segmented neutrophils (−40.1%) and basophils (−23.7%), alongside a recovery of lymphocyte (+21.6%) and monocyte (+51.1%) percentages.

### 3.2 Effects of PMPs and/or TN on immune parameters

**FIGURE 1:**
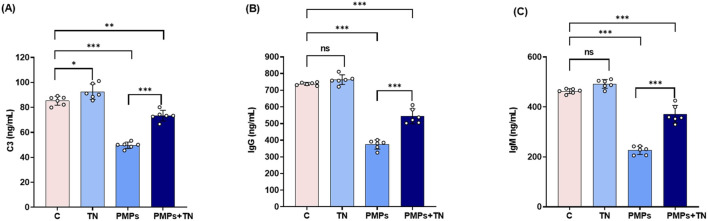
Effect of taurine (TN) and/or polystyrene microplastics (PMPs) oral dosing for 60 days on serum levels of **(A)** complement 3 (C3), **(B)** immunoglobulin G (IgG), and **(C)** immunoglobulin M (IgM) of male Swiss mice. Data are presented as mean ± SE (n = 6 per group). Statistical significance is indicated as: *** (*P* < 0.001), ** (*P* < 0.01), *(*P* < 0.05), and ns (not significant, *P* > 0.05). One-way ANOVA with Tukey’s *post hoc* test was used for comparisons.

Compared with the control group, PMPs exposure caused a significant (*P* < 0.001) reduction of serum immunoglobulins, with IgG, IgM, and C3 levels decreasing by −49.4%, −51.1%, and −41.8%, respectively (Figures 1A–C). In contrast, co-administration of TN attenuated the PMPs-induced alterations in immune parameters, significantly (*P* < 0.001) increasing IgG and IgM levels by 45.4% and 63.7%, respectively, and elevating C3 content by 47.7% compared with the PMPs group.

### 3.3 Effects of PMPs and/or TN on oxidative stress and splenic antioxidant defense

**FIGURE 2:**
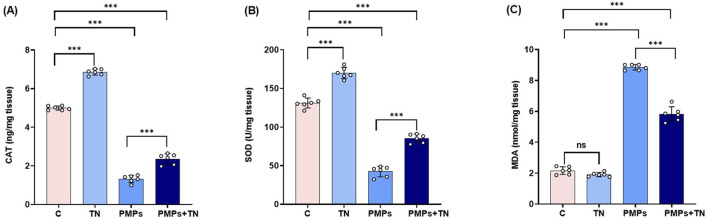
Effects of taurine (TN) and/or polystyrene microplastics (PMPs) administered orally for 60 days on splenic tissue antioxidant and oxidative stress markers in male Swiss mice: **(A)** catalase (CAT) activity, **(B)** superoxide dismutase (SOD) activity, and **(C)** malondialdehyde (MDA) content. Data are presented as mean ± SE (n = 6 per group). Statistical significance is indicated as *** (*P* < 0.001) and ns (not significant, *P* > 0.05). One-way ANOVA with Tukey’s *post hoc* test was used for comparisons.

As shown in Figures 2A,B significant (*P* < 0.001) suppression of the splenic antioxidant enzyme activities, with CAT and SOD decreasing by −73.6% and −67.7%, respectively, relative to control values. In contrast, a significant increase in splenic MDA content by **+**308.3% in the PMPs-exposed mice relative to the control one (Figure 2C). Yet, TN co-treatment significantly (*P* < 0.001) alleviated oxidative stress, restoring CAT and SOD activities by +78.8% and +101.4%, respectively, while reducing MDA levels by −34.3% compared with the PMPs group.

### 3.4 Effects of PMPs and/or TN on splenic inflammatory mediators

**FIGURE 3:**
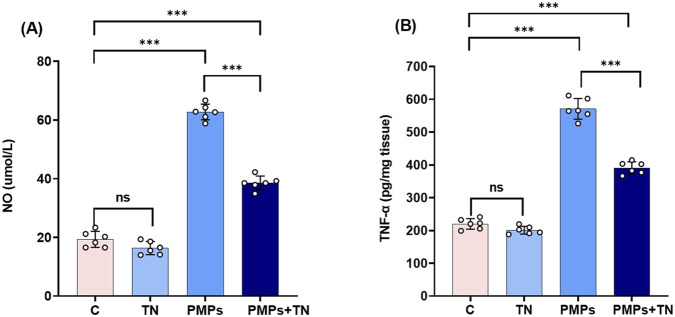
Effects of taurine (TN) and/or polystyrene microplastics (PMPs) administered orally for 60 days on splenic tissue inflammatory markers in male Swiss mice: **(A)** nitric oxide (NO) and **(B)** tumor necrosis factor-alpha (TNF-α). Data are presented as mean ± SE (n = 6 per group). Statistical significance is indicated as *** (*P* < 0.001) and ns (not significant, *P* > 0.05). One-way ANOVA with Tukey’s *post hoc* test was used for comparisons.

PMPs exposure elicited a pronounced inflammatory response, as evidenced by a significant (*P* < 0.001) elevation in splenic NO (+224.1%) and TNF-α (+159.4%) levels compared with the control group (Figures 3A,B). Taurine supplementation significantly (*P* < 0.001) reduced NO and TNF-α levels by −38.5% and −31.5%, respectively, compared with PMPs-exposed mice.

### 3.5 Effects of PMPs and/or TN on splenic histopathology

**FIGURE 4:**
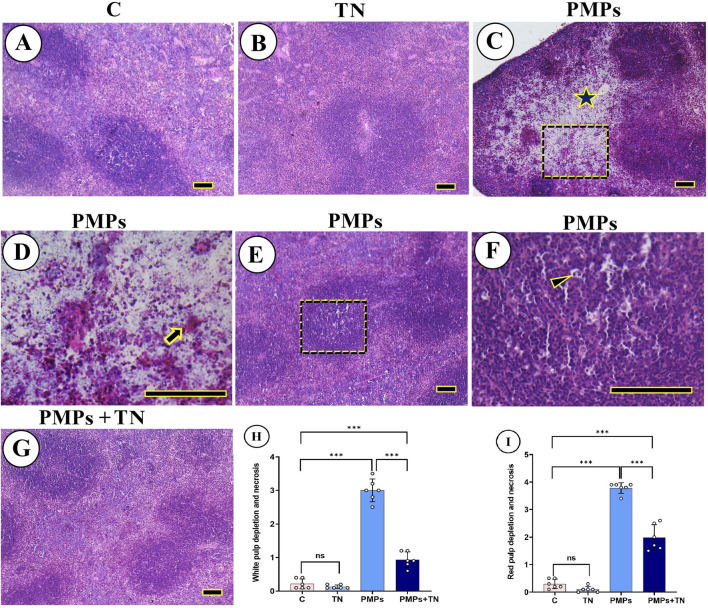
Representative photomicrographs of H&E-stained splenic tissue sections from male Swiss mice in different experimental groups: **(A)** Control, **(B)** Taurine (TN), **(C–F)** polystyrene microplastics (PMPs), and **(G)** TN + PMPs. Arrows indicate white pulp necrosis (arrowheads), necrosis in red pulp (thick black arrows), and massive depletion in red pulp (star). **(D,F)** are higher-magnification insets of the dotted squares shown in **(C,E)**, respectively. Scale bar = 50 μm. **(H,I)** Quantitative scoring of white and red pulp depletion and necrosis across the experimental groups. Data are presented as mean ± SE (n = 6 per group). Statistical significance is indicated as *** (*P* < 0.001) and ns (not significant, *P* > 0.05). Comparisons were performed using the Kruskal–Wallis test followed by Dunn’s *post hoc* test.

Histopathological examination of H&E–stained splenic sections revealed normal splenic architecture in control and TN-treated mice, characterized by well-defined white pulp and intact red pulp regions (Figures 4A,B). In contrast, mice exposed to PMPs exhibited marked structural disruption of the spleen, including massive depletion and necrosis in the red pulp and moderate necrosis in the white pulp (Figures 4C–F). Semi-quantitative lesion scoring confirmed a significant increase in both white and red pulp necrosis and depletion in the PMPs group compared with controls (Figures 4H,I). Co-administration of TN markedly ameliorated PMPs-induced splenic damage, as evidenced by reduced necrotic areas and partial restoration of splenic architecture (Figure 4G). Lesion scores for both white and red pulp were significantly decreased in the PMPs + TN group compared with PMPs alone, although they remained higher than control values.

### 3.6 Effects of PMPs and/or TN on splenic polysaccharide accumulation

**FIGURE 5:**
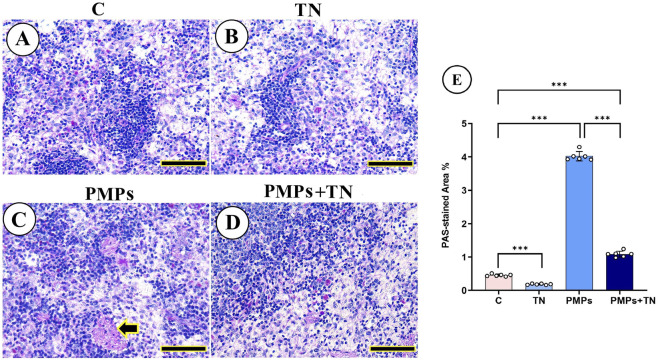
Representative photomicrographs of spleen sections stained with Periodic Acid–Schiff (PAS) from mice of different experimental groups. **(A)** Control group **(C)** showing weak PAS-positive staining. **(B)** Taurine-treated group (TN) showing minimal PAS reactivity. **(C)** Polystyrene microplastics (PMPs)-exposed group demonstrating marked elevation in PAS-positive material (Thick arrow). **(D)** PMPs + TN group showing a noticeable reduction in PAS-positive staining. Scale bar = 50 µm. **(E)** Quantitative analysis of PAS-stained area percentage in spleen sections in the different experimental groups. Data are presented as mean ± SE (n = 6 per group). Statistical significance is indicated as *** (*P* < 0.001). One-way ANOVA with Tukey’s *post hoc* test was used for comparisons.

Periodic Acid–Schiff staining of spleen sections revealed weak PAS-positive reactions in both the control and TN-treated groups, indicating minimal polysaccharide accumulation (Figures 5A,B). In contrast, the PMPs-exposed group exhibited a marked increase in PAS-positive stained areas, particularly within the splenic tissue, indicating excessive glycogen and/or glycoprotein accumulation (Figure 5C). Co-treatment with TN (PMPs + TN) noticeably reduced the intensity and distribution of PAS-positive staining compared with the PMPs group, although the values remained significantly higher than those of the control and TN groups (Figure 5D). Quantitative morphometric analysis confirmed a significant elevation in PAS-stained area percentage in the PMPs group compared with all other groups, while TN co-administration significantly (*P* < 0.001) ameliorated these alterations (Figure 5E).

### 3.7 Effects of PMPs and/or TN on splenic apoptosis

**FIGURE 6:**
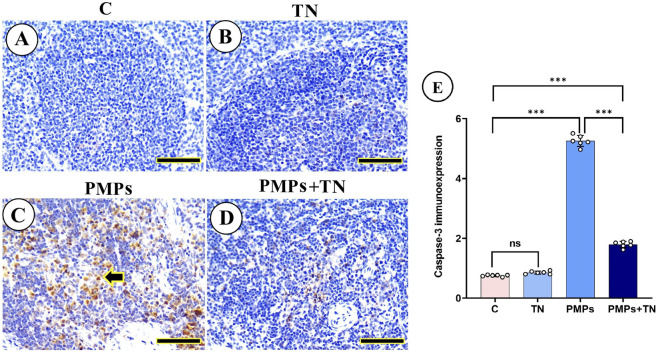
Representative photomicrograph of cleaved Caspase 3 immune-stained mice splenic tissues sections of different experimental groups. **(A)** Control group, **(B)** taurine (TN) group, **(C)** Polystyrene microplastics (PMPs) group, and **(D)** TN + PMPs group. Caspase 3-positive staining (Arrows). *Scale bar =50 μm.* **(E)** Quantification of Caspase-3-positive areas in the different experimental groups. Data are presented as mean ± SE (n = 6 per group). Statistical significance is indicated as *** (*P* < 0.001) and ns (not significant, *P* > 0.05). One-way ANOVA with Tukey’s *post hoc* test was used for comparisons.

Immunohistochemical staining for cleaved caspase-3, localized to the nuclei of apoptotic cells, revealed weak or negligible immunoreactivity in the spleens of control and TN-treated mice (Figures 6A,B). In contrast, PMPs exposure resulted in strong caspase-3–positive staining in both white and red pulp regions (Figure 6C). Quantitative analysis showed a significant increase in Caspase-3–immunopositive areas in the PMPs group compared with controls (Figure 6E). Taurine co-administration significantly reduced caspase-3 immunoreactivity relative to PMPs exposure (Figure 6D).

### 3.8 Effects of PMPs and/or TN on splenic inflammatory response

**FIGURE 7:**
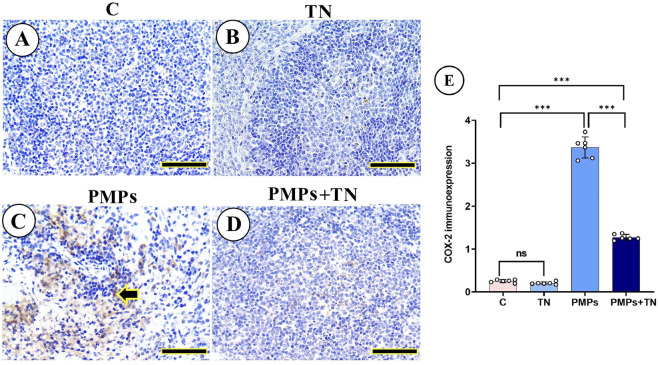
Representative photomicrograph of cleaved COX-2-immune-stained mice splenic tissues sections of different experimental groups. **(A)** Control group, **(B)** taurine (TN) group, **(C)** Polystyrene microplastics (PMPs) group, and **(D)** TN + PMPs group. COX2-positive staining (Arrows). *Scale bar =50 μm.* **(E)** Quantification of COX-2-positive areas in the different experimental groups. Data are presented as mean ± SE (n = 6 per group). Statistical significance is indicated as *** (*P* < 0.001) and ns (not significant, *P* > 0.05). One-way ANOVA with Tukey’s *post hoc* test was used for comparisons.

Immunohistochemical analysis of COX-2 expression showed minimal COX-2 immunoreactivity in control and TN-treated mice (Figures 7A,B). In contrast, PMPs exposure markedly increased COX-2–positive staining in splenic tissues, particularly within inflammatory cell populations (Figure 7C). Semi-quantitative analysis confirmed a significant elevation in COX-2–immunostained areas in the PMPs group compared with controls (Figure 7E). Taurine co-treatment significantly suppressed COX-2 expression relative to PMPs exposure alone (Figure 7D).

### 3.9 Effects of PMPs and/or TN on splenic immune- and inflammation-related gene expression

**FIGURE 8:**
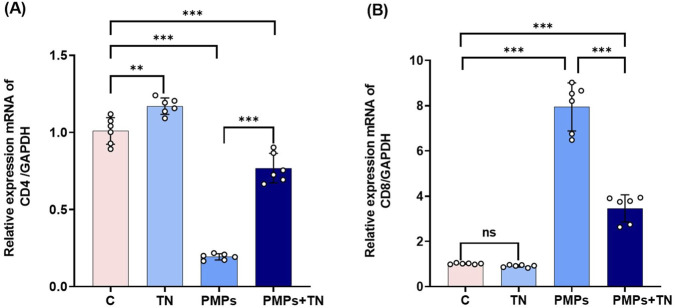
Effects of taurine (TN) and/or polystyrene microplastics (PMPs) administered orally for 60 days on mRNA expression of: **(A)** cluster of differentiation 4 (CD4) and **(B)** cluster of differentiation 8 (CD8) in the spleen of mice. GAPDH: glyceraldehyde-3-phosphate dehydrogenase. Data are presented as mean ± SE (n = 6 per group). Statistical significance is indicated as *** (*P* < 0.001), ** (*P* < 0.01), and ns (not significant, *P* > 0.05). One-way ANOVA with Tukey’s *post hoc* test was used for comparisons.

**FIGURE 9:**
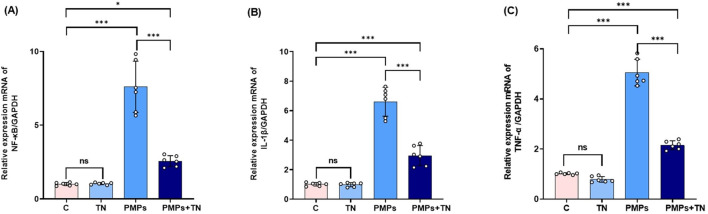
Effect of taurine (TN) and/or polystyrene microplastics (PMPs) administered orally for 60 days on mRNA expression of: **(A)** nuclear factor kappa-light-chain-enhancer of activated B cells p65 subunit (NF-κB-p65), **(B)** interleukin-1 beta (IL-1β), and **(C)** tumor necrosis factor alpha (TNF-α) in the spleen of mice. GAPDH: Glyceraldehyde-3-phosphate dehydrogenase. Data are presented as mean ± SE (n = 6 per group). Statistical significance is indicated as: *** (*P* < 0.001), *(*P* < 0.05), and ns (not significant, *P* > 0.05). One-way ANOVA with Tukey’s *post hoc* test was used for comparisons.

PMPs exposure markedly impaired adaptive immune signaling, as reflected by significant (*P* < 0.001) downregulation of splenic CD4 (−80.9%) but upregulation of CD8 (+682.6%) mRNA expression compared with controls (Figures 8A,B). Conversely, PMPs strongly upregulated pro-inflammatory gene expression, with NF-κB-p65, IL-1β, and TNF-α increasing by +654.0%, +552.3%, and +396.7%, respectively (Figures 9A–C). In contrast, TN co-administration significantly restored immune gene expression, increasing CD4 (+298.2%) and decreasing CD8 (+56.5%) levels relative to PMPs exposure. In parallel, TN markedly suppressed inflammatory signaling, reducing NF-κB-p65, IL-1β, and TNF-α expression by −66.2%, −57.4%, and −55.3%, respectively.

### 3.10 Effects of PMPs and/or TN on splenic apoptosis-related gene expression

**FIGURE 10:**
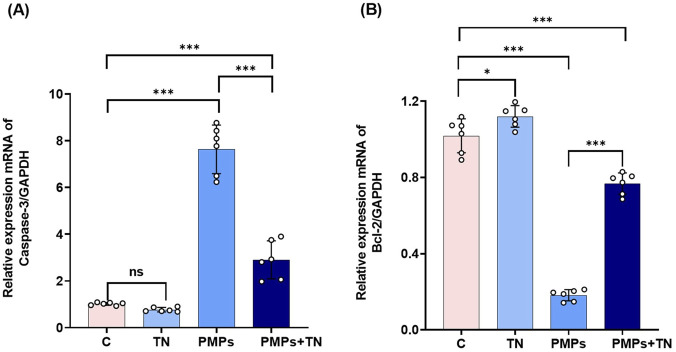
Effect of taurine (TN) and/or polystyrene microplastics (PMPs) administered orally for 60 days on mRNA expression of: **(A)** Caspase-3 and **(B)** B-cell lymphoma-2 in the spleen of mice (BCL-2). GAPDH: Glyceraldehyde-3-phosphate dehydrogenase. Data are presented as mean ± SE (n = 6 per group). Statistical significance is indicated as: *** (*P* < 0.001), *(*P* < 0.05), and ns (not significant, *P* > 0.05). One-way ANOVA with Tukey’s *post hoc* test was used for comparisons.

As shown in the Figures 10A,B, PMPs exposure induced a strong pro-apoptotic shift in splenic tissue, evidenced by a significant (*P* < 0.001) upregulation of caspase-3 expression (+647.7%) and a concomitant significant (*P* < 0.001) downregulation of the anti-apoptotic gene BCL-2 (−82.1%) compared with control mice. Taurine co-treatment significantly counteracted these effects (*P* < 0.001), reducing caspase-3 expression by −61.9% and increasing BCL-2 expression by +322.0% relative to the PMPs group.

### 3.11 Correlation between hematological parameters, immune regulation, inflammation, apoptosis, and oxidative stress in splenic tissue

**FIGURE 11:**
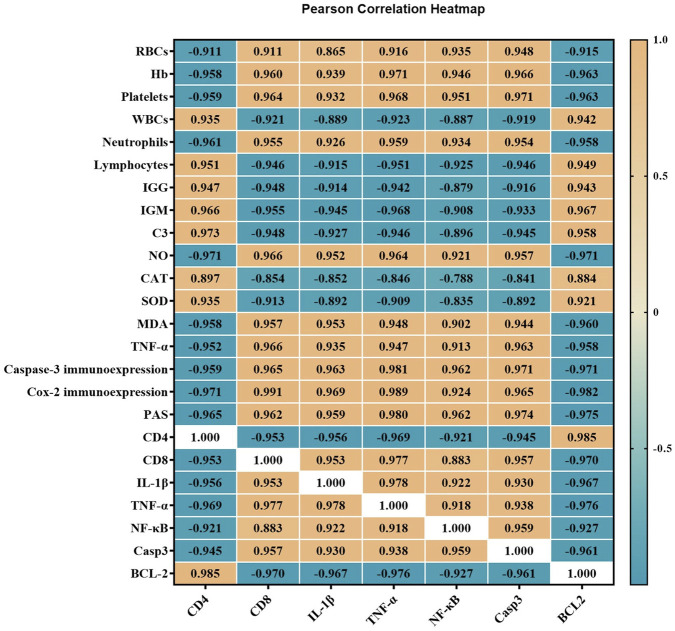
Pearson correlation heatmap illustrating the relationships among hematological indices, immune cell markers, oxidative stress parameters, antioxidant enzymes, and Caspase-3 and COX-2 immunoexpression, as well as the expression of inflammatory cytokines, apoptotic markers, and immune-regulatory genes in the splenic tissue across the experimental groups. The analyzed variables include red blood cells (RBCs), hemoglobin (Hb), platelets, white blood cells (WBCs), neutrophils, lymphocytes, immunoglobulin G (IgG), immunoglobulin M (IgM), complement component 3 (C3), nitric oxide (NO), catalase (CAT), superoxide dismutase (SOD), malondialdehyde (MDA), tumor necrosis factor-alpha (TNF-α), interleukin-1β (IL-1β), Caspase-3, cyclooxygenase-2 (Cox-2) immunoexpression, B-cell lymphoma 2 (BCL-2), CD4 and CD8 T lymphocytes, and periodic acid–Schiff (PAS)-stained area. Correlation coefficients (r) range from −1 to +1, where +1 indicates a perfect positive correlation, −1 indicates a perfect negative correlation, and 0 denotes no correlation. The color gradient represents the strength and direction of correlations, with orange indicating positive correlations and blue indicating negative correlations. Values within each cell represent the Pearson correlation coefficient between corresponding variables.

The Pearson correlation heatmap revealed strong interrelationships between hematological indices, immune cell markers, oxidative stress parameters, antioxidant enzymes, and Caspase-3 and COX-2 immunoexpression, as well as the expression of inflammatory cytokines, apoptotic markers, and immune-regulatory genes (Figure 11). Hematological parameters, including RBCs, Hb, platelets, and neutrophils, showed strong positive correlations with the gene expression of CD8, pro-inflammatory cytokines (IL-1β and TNF-α), NF-κB, and the apoptotic marker Caspase-3. In contrast, these hematological indices exhibited strong negative correlations with the gene expression of the anti-apoptotic marker BCL-2 and the immune-regulatory marker CD4. On the other hand, WBCs and lymphocytes showed positive correlations with the gene expression of BCL-2 and CD4, while exhibiting negative correlations with CD8, IL-1β, TNF-α, NF-κB, and Caspase-3.

Pro-inflammatory cytokines (IL-1β and TNF-α) and NF-κB gene expression were highly positively intercorrelated and also positively associated with Caspase-3 gene expression together with Caspase-3 and COX-2 immunoexpression, reflecting a tightly linked inflammatory–apoptotic axis. In contrast, BCL-2 gene expression showed negative correlations with these inflammatory and apoptotic markers. Histopathological indices, including PAS-stained area, were positively associated with the gene expression of pro-inflammatory cytokines, NF-κB, and Caspase-3, while showing inverse correlations with BCL-2 gene expression, thereby linking structural tissue alterations to coordinated inflammatory and apoptotic responses.

At the molecular level, the immune-regulatory genes CD4 and CD8 were negatively correlated with each other. CD4 gene expression exhibited strong negative correlations with the gene expression of TNF-α, IL-1β, NF-κB, and Caspase-3, as well as with Caspase-3 and COX-2 immunoexpression, while showing a positive association with BCL-2 gene expression. In contrast, CD8 gene expression demonstrated the opposite correlation pattern.

## 4 Discussion

The present findings demonstrated that chronic oral exposure to PMPs induced significant alterations in hematological homeostasis, affecting both erythroid and leukoid lineages, thereby reflecting systemic toxicity and immune alterations. Hematological indices are highly sensitive biomarkers of toxic exposure (Gwaltney-Brant, 2019), and the observed alterations provide early evidence of PMPs-induced stress responses and inflammatory activation. PMPs exposure resulted in marked erythrocytosis, as evidenced by significant elevations in RBC count, Hb concentration, and HCT values. Similar findings were reported by Wang L. et al. (2022), who observed increased RBCs, Hb, HCT, and red cell distribution width following oral exposure to 2 mg PMPs/kg/day 3 times a week for 5 weeks, indicative of a polycythemia vera–like condition (Yan et al., 2012). In support of this, Wang L. et al. (2022) demonstrated that delayed increases in reticulocyte percentage following PMPs exposure reflected effective compensatory erythropoiesis in the bone marrow. Microplastics have been also reported to impair oxygen diffusion, induce vascular inflammation, and disrupt mitochondrial respiration, all of which may stimulate erythropoietin release and enhance erythropoiesis (Fu et al., 2024; Huo et al., 2024; Lomonaco et al., 2024). However, these erythrogram changes may reflect hemoconcentration, stress-related shifts, or compensatory responses to tissue hypoxia and oxidative stress rather than a direct stimulatory effect on erythropoiesis (Haase, 2010). The concomitant elevation in MCHC observed in the present study further suggests alterations in erythrocyte membrane integrity and Hb packing, potentially driven by oxidative injury. In this context, MPs have been shown to destabilize lipid membranes of human RBCs by inducing mechanical stretch (Fleury and Baulin, 2021). In parallel, PMPs-exposed mice exhibited pronounced thrombocytosis, indicative of heightened megakaryocytic activity and platelet release. Consistent with these findings, exposure to 5-µm PMPs increased platelet counts and MCV in mice (Sun et al., 2021), while PMPs have also been shown to enhance platelet activation and aggregation in zebrafish (Gao et al., 2021). Reactive thrombocytosis is commonly associated with systemic inflammation and oxidative stress (Gaman et al., 2019), both of which were evident in the present study. Correlation analysis indicated that platelets were positively associated with pro-inflammatory cytokines (TNF-α and IL-1β), further linking thrombocytosis to the inflammatory response induced by PMPs. Activated platelets act not only as hemostatic elements but also as inflammatory mediators by releasing cytokines and interacting with leukocytes, thereby amplifying immune alterations and inflammatory responses (Sonmez and Sonmez, 2017). Accordingly, the excessive platelet elevation observed here suggests the development of a pro-thrombotic and pro-inflammatory state following PMPs exposure.

Leukogram analysis revealed leukocyte redistribution and altered immune cell profiles, characterized by reduced total WBC counts, accompanied by neutrophilia and basophilia, alongside lymphopenia and monocytopenia. A concentration-dependent reduction in WBC counts has similarly been reported in C57BL/6 mice exposed to 6–600 μg PMPs/day for 15 days (Abdel-Zaher et al., 2023). These patterns likely reflect stress- and inflammation-driven shifts in leukocyte distribution. Neutrophilia is a hallmark of acute and chronic inflammation and may result from enhanced bone marrow release or demargination under cytokine stimulation (Castanheira and Kubes, 2019). Microplastics have been shown to promote neutrophil migration, ROS production, and pro-inflammatory cytokine release through mechanisms involving histone lactylation and toll-like receptor pathways (Wang et al., 2025). Additionally, MPs can be phagocytosed by neutrophils, leading to cell death and further inflammatory amplification (Park et al., 2024). The systemic distribution of MPs allows interactions with neutrophils across multiple organs, exacerbating immune alterations (Giustarini et al., 2025). Basophilia, although less frequently reported, may indicate hypersensitivity-like responses to particulate exposure (Liu et al., 2025). Conversely, the reductions in lymphocytes and monocytes suggest potentially altered adaptive and innate immune responses (Bieber and Autenrieth, 2015; Finfer et al., 2023). Pearson correlation analysis showed that lymphocyte counts were positively correlated with CD4 expression and BCL-2, while neutrophil counts were positively associated with pro-inflammatory markers (TNF-α, IL-1β, and NF-κB) and Caspase-3, highlighting the obvious association between immune cell redistribution, inflammation, and apoptosis. Lymphopenia may result from oxidative injury, apoptosis, or redistribution to inflamed tissues, whereas monocytopenia may reflect suppressed myelopoiesis or excessive differentiation into tissue macrophages (Finfer et al., 2023). Collectively, these hematological changes highlight systemic stress and immune perturbations associated with PMPs exposure.

Notably, TN co-administration markedly ameliorated PMPs-induced hematological disturbances. Taurine significantly reduced erythrocytosis and improved Hb and HCT levels, suggesting attenuation of hypoxic stress and oxidative injury. Taurine is known to protect erythrocytes against oxidative hemolysis, regulate ion homeostasis, and preserve membrane fluidity, which likely underlies its corrective effects on red blood cell parameters (Bertolone et al., 2020; Ali et al., 2022). Taurine also significantly reduced PMPs-induced thrombocytosis, reflecting its ability to suppress inflammation and platelet overactivation (McCarty, 2004; Santhakumar et al., 2012). Platelets avidly concentrate TN, and, for reasons not yet clear, this TN tends to blunt the calcium influx triggered by pro-aggregant agonists, thereby rendering platelets more stable (Elizarova, 1996). This effect may contribute to reducing the thrombo-inflammatory risk associated with chronic PMPs exposure. In the leukogram, TN restored total WBC counts and corrected leukocyte imbalances by reducing neutrophilia and basophilia while promoting recovery of lymphocyte and monocyte populations. Taurine has been shown to decrease the expression of pro-inflammatory cytokines such as TNF-α and IL-1β in neutrophils and monocytes, which can reduce inflammation and potentially mitigate leukocytosis and neutrophilia (Ma Y. et al., 2022). Tau-Cl, a derivative of TN, inhibits the release of myeloperoxidase from neutrophils, reducing oxidative damage and inflammation (Kim et al., 2020). Taurine’s role in lymphocyte proliferation indicates it might help in managing lymphocyte levels, potentially alleviating lymphocytopenia (Fazzino et al., 2010).

One of the most notable findings was the marked suppression of humoral immune parameters following PMPs exposure, as evidenced by significant reductions in serum IgG, IgM, and C3 levels. Immunoglobulins are essential mediators of adaptive immunity (Mix et al., 2006), while C3 plays a pivotal role in opsonization, immune complex clearance, and innate-adaptive immune crosstalk (Toapanta and Ross, 2006). The decline in these parameters suggests compromised B-cell function and antibody-mediated defense, potentially increasing susceptibility to infection. Supporting this interpretation, oral MP ingestion in mice increased macrophage and natural killer -cell infiltration while reducing B-cell infiltration in hepatic tissues (Zhao et al., 2021), indicating altered immune cell distribution. Moreover, MPs can disrupt lymphoid organ integrity, interfere with antigen presentation, and dysregulate immune cell differentiation (Wolff et al., 2023; Li, 2025). The concomitant reduction in C3 may also reflect sequestration of complement and immunoglobulin proteins within a protein biocorona formed on MP surfaces following their entry into the bloodstream (Lundqvist et al., 2008), further exacerbating immune perturbations.

Notably, TN co-treatment effectively restored IgG, IgM, and C3 levels toward physiological values, underscoring its capacity to preserve humoral immunity under toxic insult. Taurine is known to stabilize immune cell membranes, support B-cell maturation, and regulate cytokine signaling, which may collectively account for the observed recovery. Taurine influences the expression of immune-related genes in macrophages, significantly increasing the mRNA levels of chemokine receptors such as CXCR2 and CXCR4, which are crucial for immune cell function (Satsu et al., 2022). Song et al. (2023) reported that TN enhances B-cell receptor-mediated signal transduction, leading to increased activation of B cells and higher IgG production, which is essential for adaptive immunity.

Oxidative stress emerged as a central mechanism underlying PMPs-induced splenic toxicity (Guo et al., 2024). The spleen is particularly vulnerable due to its high cellular turnover and dense immune cell population (Lewis et al., 2019). PMPs exposure markedly increased splenic MDA levels while significantly suppressing CAT and SOD activities, suggesting excessive ROS generation and compromised antioxidant defenses. This imbalance likely results from increased ROS production and direct inhibition or consumption of antioxidant enzymes during PMPs metabolism (Wang Y. L. et al., 2021; Lin et al., 2024). Consistent with the histochemical findings, the marked elevation in PAS-positive material within the splenic tissue may be attributed to oxidative stress-mediated disturbances in carbohydrate metabolism and inflammatory tissue injury, leading to the accumulation of glycogen, glycoproteins, and other polysaccharide-rich substances within damaged splenic cells and extracellular components (Djouina et al., 2023). Oxidative stress was accompanied by a significant inflammatory response, as indicated by elevated NO and TNF-α levels and increased COX-2, a key enzyme involved in prostaglandin synthesis and inflammatory amplification (Rajakariar et al., 2006), and immunoexpression within splenic inflammatory cells. Excessive NO production exacerbates oxidative and nitrosative damage, while TNF-α amplifies immune activation and tissue injury. At the molecular level, PMPs strongly activated inflammatory gene networks, including IL-1β, TNF-α, and NF-κB-p65, confirming the establishment of a persistent pro-inflammatory response. This inflammatory burden was closely linked to apoptosis, as evidenced by increased cleaved caspase-3 immunoreactivity and a marked shift in apoptosis-related gene expression, characterized by caspase-3 upregulation and BCL-2 downregulation. This mitochondrial-mediated apoptotic cascade likely contributes to lymphocyte depletion and splenic architectural damage observed histologically.

In contrast, TN co-administration substantially attenuated lipid peroxidation while restoring antioxidant enzyme activities, highlighting its role as a direct free radical scavenger and an indirect enhancer of endogenous antioxidant systems. Taurine has been widely documented as a multifunctional cytoprotective molecule with significant antioxidant properties. Its protective effects are attributed to several complementary mechanisms, including maintenance of intracellular ionic balance, preservation of membrane structural stability, and direct neutralization of ROS (Biasetti and Dawson Jr, 2002). Moreover, TN has been reported to facilitate the transformation of GSH into S-nitrosoglutathione, a derivative with enhanced antioxidant potential compared to GSH alone (Chiueh and Rauhala, 1999). TN has also been reported to exert antioxidant effects by modulating cellular antioxidant defense systems and reducing excessive ROS, including superoxide anions and hydroxyl radicals, thereby protecting cells against oxidative damage (Shen et al., 2024). At the molecular level, TN influences redox-sensitive transcriptional pathways, including Nrf2 and NF-κB, leading to upregulation of endogenous antioxidant defenses (Schaffer et al., 2009; Ghanim et al., 2022). In addition, TN contributes to the preservation of mitochondrial structural and functional integrity, which limits excessive ROS production at its principal source and supports the maintenance of cellular homeostasis (Jong et al., 2012). Of note, the significant elevation of antioxidant enzyme activities in the TN-only group may reflect an adaptive enhancement of antioxidant capacity, further supporting its role in maintaining oxidative balance. Taurine significantly suppressed NO, TNF-α, and COX-2 expression, reinforcing its anti-inflammatory efficacy. This suppression may be mediated through inhibition of NF-κB activation, a hypothesis further supported by gene expression data. TauCl, has been shown to inhibit the activation of NF-κB, a key regulator of inflammatory responses, thereby reducing the production of pro-inflammatory cytokines (Schuller-Levis and Park, 2004). TauCl also plays a role in resolving inflammation by promoting the production of antioxidant proteins and reducing oxidative stress (Kim and Cha, 2014). Taurine markedly reduced macrophage infiltration and preserved splenic architecture, suggesting that it limits PMPs-induced immune cell overactivation and tissue injury. Taurine effectively counteracted this pro-apoptotic shift by suppressing caspase-3 expression and restoring BCL-2 levels, thereby promoting cell survival. These anti-apoptotic effects may stem from TN’s ability to stabilize mitochondrial membranes (Lambuk et al., 2019), reduce oxidative stress, and inhibit inflammatory responses (Ma J. et al., 2022).

At the molecular level, CD4 and CD8 gene expression were negatively correlated. CD4 expression showed negative correlations with TNF-α, IL-1β, NF-κB, Caspase-3 gene expression, and Caspase-3 and COX-2 immunoexpression, while being positively associated with BCL-2 expression. In contrast, CD8 exhibited the opposite pattern, indicating that T-cell imbalance contributes to splenic inflammatory and apoptotic responses. The marked downregulation of CD4 accompanied by upregulation of CD8 suggests an imbalance in T-cell subpopulations, reflecting disrupted adaptive immune regulation and enhanced cytotoxic/inflammatory responses within splenic tissue (Cabrera-Perez et al., 2014). Similarly, the administration of 0.1 and 1 mg/kg b.wt doses of MPs for 10 weeks led to a remarkable decrease in the CD4^+^/CD8^+^ cell ratio in mice (Chen et al., 2024). Such skewing may favor cytotoxic responses at the expense of immune regulation and antibody production. Simultaneously, PMPs strongly activated inflammatory gene networks, including IL-1β, TNF-α, and NF-κB-p65, confirming the establishment of a chronic pro-inflammatory milieu. NF-κB is a central transcription factor linking oxidative stress to inflammation and apoptosis (Lingappan, 2018; Nna et al., 2019), and its activation likely orchestrates many of the downstream pathological events observed in this study. Taurine co-administration effectively improved CD4 and CD8 expression and suppressed inflammatory gene activation, further supporting its role as a key modulator of immune homeostasis.

## 5 Limitations and future directions

Despite the valuable insights provided by this study, several limitations should be acknowledged. The first limitation of this study is that only male mice were used. This choice was made to minimize potential variability that could arise from hormonal fluctuations in females, which may affect immune responses and related molecular markers (Sciarra et al., 2023). Nevertheless, males and females are known to differ in both innate and adaptive immunity, but such differences do not inherently increase variability in experimental results (Klein and Flanagan, 2016). Therefore, our results may not fully apply to female subjects. Future research should include both sexes to explore possible sex-specific effects of PMPs on immune function and the immunomodulatory potential of TN. The second limitation of this study is that, although mouse models allow for controlled investigation of the mechanisms underlying PMPs–induced toxicity, the results may not directly translate to humans due to species-specific differences in exposure conditions, physiology, and metabolism. Hence, complementary epidemiological studies are necessary to more precisely evaluate the probable health hazards related to PMPs exposure in humans. The third limitation is that this study employed only a single polymer type and particle size range, a single oral dose, and focused primarily on spleen-centered endpoints. The single-dose design was chosen as an early-stage exploratory investigation to provide a controlled assessment of immunotoxicity while minimizing animal use, in line with the 3Rs ethical principles (Reduce, Refine, and Replace). This approach allows for mechanistic insights while ethically limiting animal numbers, though it may constrain the ability to fully characterize the dose-response relationship. Despite this, these choices may limit the generalizability of the findings to other polymers, exposure regimens, or immune organs, and future studies should expand these variables to confirm and extend our results. The fourth limitation is that our study primarily relied on qPCR and immunohistochemistry to evaluate pathway involvement, which provides only indirect evidence of the underlying molecular mechanisms. To more definitively identify TN’s molecular targets and validate the implicated signaling pathways, future studies should incorporate direct protein measurements (e.g., Western blotting), functional assays, and approaches such as pathway inhibition or gene silencing. Besides, given that adaptive immune function was assessed only by splenic CD4 and CD8 mRNA expression, further studies employing more detailed phenotypic analyses, such as flow cytometry of T-cell subsets, are necessary to comprehensively characterize immune alterations. Finally, a fifth limitation is the absence of a positive/active control in this study, which limits direct comparison with established immunoprotective agents. Although our primary aim was to evaluate the independent protective potential of TN against PMPs-induced immunotoxicity, future studies should include a standard immunoprotective or anti-inflammatory drug to benchmark TN’s efficacy and allow for more comprehensive contextualization of the results.

## 6 Conclusion

Exposure to PMPs induces hematological and immune disturbances, including erythrocytosis, thrombocytosis, leukopenia, and altered leukocyte distribution, reflecting a systemic inflammatory and immunosuppressive state. These changes are accompanied by oxidative stress and alterations of inflammatory response and apoptotic reactions, resulting in splenic injury. Taurine supplementation attenuated these adverse effects, restoring hematological balance, preserving splenic architecture, and supporting immune function. Notably, the study employed a single, physiologically relevant dose of TN (200 mg/kg b.wt) as an early-stage exploratory investigation, consistent with the 3Rs principles (Reduce, Refine, and Replace), to ethically minimize animal use while obtaining mechanistic insights. Overall, the findings highlight the spleen as a primary target of PMPs-induced immunotoxicity and suggest that TN may exert a protective role against PMPs-related immune and inflammatory alterations, warranting further studies to elucidate the underlying molecular mechanisms and dose-response relationships.

## Data Availability

The original contributions presented in the study are included in the article/supplementary material, further inquiries can be directed to the corresponding author.
